# Supplementary material for: Patterns of Intron Gain and Loss in Fungi
Source: PLoS Biol. 2004 Nov 30;2(12):e422. doi: 10.1371/journal.pbio.0020422 (PMC532390; doi:10.1371/journal.pbio.0020422)
Supplement: Table S1 — Also available at http://genes.mit.edu/NielsenEtAl/. (4.3 MB ZIP). [file pbio.0020422.st001.zip › NielsenEtAl/html/255.html]

AN4892.1.NCU09435.1.MG03265.1.FG00349.1


```
 CLUSTAL W (1.82) Multiple Sequence Alignments - Introns Inserted


Sequence 1: NCU09435.1	1167 aa
Sequence 2: MG03265.1	1057 aa
Sequence 3: FG00349.1	997 aa
Sequence 4: AN4892.1	1141 aa
Alignment Length: 1253 aa
Number Identitical Residues: 282 aa
Alignment Score (without introns) 16289


MG03265.1 	---~----------------------~-------------------------~-------
NCU09435.1	---~----------------------~-------------------------~-------
FG00349.1 	---~----------------------~-------------------------~-------
AN4892.1  	MMG1IEAPYEVFTEGRFAWRGRKTAG2TRPPSDRRRAPRVERIILALTAFIT2SNLCRLL
          	  .  .:. .  :..  :  . .::. : ..:.   :.  .    : ::  : :. .   

MG03265.1 	----~-----MASDVG-------------DAQQQPGWDHDSTEDGPD--GLQSTAQHSDS
NCU09435.1	----~-----MSDDYDPTNINAASWEEQEDYGEADGSEQYGEAEGSEQQDIQHSSEHQEA
FG00349.1 	----~--------------------------MASEGTSWGAEDIGAQGDQVQHSEEQVDN
AN4892.1  	LESL2SIPIFMAEDDAEKAFFEAQAMNAGSMGYNGAEDQNANASDSDEYDPSSTLQDQYP
          	 .:  : .   :..   .    :.  .  .     . .  .   ..:    . : :.   

MG03265.1 	NVQNQ----QLQATSVAHGDSEDA-AEYDPD-----SVPVAITSPKAKPLKPSPQPAA--
NCU09435.1	EYSVESNPNDLGDHPSQDGNTDDVGDDYDPASVVTSSVPAPPSAPAQEDSKTAPQPAAPV
FG00349.1 	YAHDS---LASGDADAADNGTEDDGGEYDPE-----SVTIGTPAMVPEPASSGTQRQT--
AN4892.1  	APSENLQLQETDTPTSVSQPQPSFREDADPAG--NAHPSQPPSRPESQASTSVPATGVSV
          	    . .               .   : ** .  .:  .   .    :  .. .   .. 

MG03265.1 	AKKRKTAGGFLVGDSDDSEDEN--------------------------------------
NCU09435.1	AKKPRTAGGFLVGDSDDEDEDGDDDGEPQQQQQQQQQQQPHQVVHKETGSGASSSGGSAP
FG00349.1 	-SKPKMSGGFIVEASDDEDEDE--------------------------------------
AN4892.1  	QPKTRTIGGFVVEDEDEDDAGDADYEPPAVLGVEDMNIIPSQPIPG--NANEATSTPDVS
          	  * :  ***:*  .*:.:    .   .     .. .  . .      .:. :::  . .

MG03265.1 	-PTVASVQMP--DLKPKPESSPVPQNLNGGAQVSESSSGQGAQSHGS-----------NQ
NCU09435.1	APASASATAAPQSHSPAPQTATLTVQDNAGATTFNAPPVPQQVSHQSGATTAAVPTTPSS
FG00349.1 	-DEDEDEDEDEQPASAVPQTDAVSTQNHPGPSTTSDEHVPAAPTHAP----------VPP
AN4892.1  	LDEAAQESASAKNVPNSSLSSVSLAFKNDGSMDLGQNLYNSRTSLQPEIARESATATPAP
          	     .     .     . :       : *.            :  .  :  : .::   

MG03265.1 	SHPSVVPSVLSH---DPIALLELRVKEDPRGAMDSWLSLIAAYRQRNNIEQCRDVFERFL
NCU09435.1	AAPAVDPTPVTQPAPDRVAIYEDQIRDDPRGAMNAWLELMKEKRARNDIDGARQVYERFL
FG00349.1 	AVPSNVTPVLPG--FGPVDLLEFRVKEDPRGDMDAWQELIASHRDFSPLEKARSTYNRFV
AN4892.1  	DSPSTAKGRLPH---DRVGILEDRIREDPRGDIPAWLELINEHRSRNRIDSARDVYERFL
          	  *:     :.    . : : * ::::**** : :* .*:   *  . :: .*..::**:

MG03265.1 	AVFPHA0AEVWVQYADMELSQGNFVEAEAIFGKSLMSVPNVQLWTVYLDYIRRRNDLN-D
NCU09435.1	AIFPQA0ADIWVEYLDLELSLNNFPQAEGIFAKCLMTTPNVNLWTRYLDYIRRRNDLN-D
FG00349.1 	EIFPQA0ADKWVEWIELELKYNNFVEVEQLFGRCLMQVPNVKLWTVYLDYIRRRNDLNND
AN4892.1  	KVFPLS0AEMWVAYATMESELNELFRLEQIFNRTLLTIPAVQLWTVYLDYVRRRNPLSTD
          	 :** : *: ** :  :* . .:: . * :* : *:  * *:*** ****:**** *..*

MG03265.1 	SSGRARQVVTQAYDFVIDNVGLDKDASKIWNDYIQFIRLAPGVVGGSAWQDQQKMDQLRK
NCU09435.1	STGQARQTVSQAYEFVIDNIGLDKDSGKIWAEYIQFIKFGPGTVGGSQWQDQQKMDQLRK
FG00349.1 	PSGQARRTVTQSYEFVIDNIGVDRDSGNIWQQYVQFVKNGPGQIDGTDWQDRQKMDQLRG
AN4892.1  	TTGQARKVISSAYELALQHIGMDKESGSIWADYIQFIRSGPGNVGGSGWQDQQKMDLLRK
          	.:*:**:.::.:*::.::::*:*:::..** :*:**:: .** :.*: ***:**** ** 

MG03265.1 	AYQRAVCIPLSNVNTLWREYDQFEKGLNPTT~GRKYLNERSPAYMTAKSANTALENIMRN
NCU09435.1	AYQRAICVPISNVNTLWKEYDQFEMGLNKLT~GRKYLSEKSPSYMSAKSANTALEHITRG
FG00349.1 	IYRRAVAVPMSTVNNLWKEYDQFEMGLNKMT0GRKFIQERSPVYMSAKSANIALDNITRH
AN4892.1  	AYQRAICVPMQAVNTLWKEYDQFEMGLNKLT0GRKFLQEQSPSYMTARSSYTELQNFTRD
          	 *:**:.:*:. **.**:****** ***  * ***::.*:** **:*:*:   *::: * 

MG03265.1 	LVRTTLPRLPPAPGFEGDVEFAEQVDLWKRWVKWEKEDPLDLATD--DPELFKKRILYAY
NCU09435.1	LNRTNLPRLPPAPGFDGDQEFMEQVEIWKKWIAWEKSDPLDLKDDKDQPGLYQKRILYVY
FG00349.1 	LDRTNLPRLPPAPGFNGDQEFRDQVEMWKKWIAWEKEDPLVLKSD--EPKAYNQRVLHVY
AN4892.1  	LNRTTLPRLPPVPGSEGDFEYLQQIEIWKRWINWEKGDPLVLKED--DLTAYKGRVVYVY
          	* **.******.** :** *: :*:::**:*: *** *** *  *  :   :: *:::.*

MG03265.1 	KQAIMALRFCPEIWVDAAEWCFEN-------SILVNGKDAGLEFLTEGIEANPESVLLAL
NCU09435.1	NQALMALRFWPEMWVDAAQWCFDNNITTVENKVTKDGNANGVEFLIRGIEANPESVLLAF
FG00349.1 	KQALMALRFWPEIWVDAAEWCFQN-------DIRENDKEMGTELLVEGIKANRESVLLAL
AN4892.1  	KQALMALRFLPEIWFEAADFCFLN-----------DMETEGNEFLKNGIDANPESCLLAF
          	:**:***** **:*.:**::** *           : :  * *:* .**.** ** ***:

MG03265.1 	KHADRIETTFPVGEGDDAKIERGNAVKAPYNKLLDTLYGQIKTLKEKEKAEIVKLRESAP
NCU09435.1	KHADHIESTYPIEENDEAKIQRGEAVKAPYNKVLDTLYAMIKSLKEKEAAQIAKLQEMTA
FG00349.1 	KHADHIEVNYPDKEVDKAEFAQ--AVRKPYDDVLETLYEMGDKVKEREKLEISTLKQAAA
AN4892.1  	KRADRLEITS---ESEQDPIKRGAKVREPYDRLLDALYDLIAKARTREAQDVARLEETFK
          	*:**::* .    * :.  : :.  *: **: :*::**    . : :*  ::  *.:   

MG03265.1 	SVPEH---EIEEGEDCAN---PMPSELEEKISALEKGFGAQTDLLARTVSFVWIALARAM
NCU09435.1	AQESK---AGSDNEDGDG---AADNIKKAPIEAIQKGYAAQTQLLSRMISFVWIALIRAM
FG00349.1 	QDPVQTSIEENDDDEDNT---PKRSPTEERILAIQKGYAAETQLLSRTISYVWIALARAM
AN4892.1  	LRPDTQPAANDDDDDDQSETKAKESVKNAQIEAVRHAHSIQIGILSKTVSFAWIALMRAM
          	      .   .:.::   .:..  .  :  * *:.:... :  :*:: :*:.**** ***

MG03265.1 	RRIQGKGQPGSPMGGMRQVFSEARKRGRLTSDVYVAIAQLEWTVYKDPSGGKIFDRGSKL
NCU09435.1	RRVQGKGGLNVPLGGMRKAFHDARARGRLTSDVYAAVAQLEWTIYKDPAGGKIFDRGAKL
FG00349.1 	RRIQGKG--SQAEGGLRKVFTDARQKGRLTSDVYVAVALLESVVYKDPVGAKIFERGARL
AN4892.1  	RRIQGKGKPGEVPG-SRQVFADARKRGRITSDVYIASALIEYHCYKDPAATKIFERGAKL
          	**:****  .   *  *:.* :** :**:***** * * :*   **** . ***:**::*

MG03265.1 	FPEDEIFMLEYLKYLHSREDTTN1ARVVFETCVNKLTQNPATVHKAKPLYSYFHKYESKF
NCU09435.1	FPEDENFTLENIKYLHSRDDHTN~ARVLFETVVNRLTQKPELVHKAKPLYQYFHKYESQF
FG00349.1 	FPNDEMFMIEYLKYLHSKDDTTN1ARVVFETCINRLVSNPDTLAKAKLLYAYFHKYESQY
AN4892.1  	FPEDENFALEYLKHLIDINDIIN1ARAVFEMTVRKLAANPENVHKTKPIFAFLHEYESRY
          	**:** * :* :*:* . :*  * **.:**  :.:*. :*  : *:* :: ::*:***::

MG03265.1 	GELSQTAKLEKRMAELFPEDPSLAHFSARFMSDRFDPIAARIIVSPAAQLRPK-MVMPSI
NCU09435.1	GELAQVTKLEKRMAELFPEDPKLAAFTARYASDKFDPITARIIVSPTTQLRPKNMIMPSI
FG00349.1 	GELSQISKLEDRMAELFPEDPKLKSFVDRFSTEKFDPIATPIIISKTAQMRPK-QIVPVV
AN4892.1  	GDLVQVINLETRMRELFPDDPTLEQFAHRFSAPNFDPTTFRPIISP-SQTRPK-TVYPGG
          	*:* *  :** ** ****:**.*  *  *: : .*** :   *:*  :* ***  : *  

MG03265.1 	EAN------------ARAGSPM-IRPLNSPKVG--FLQAVNSPKRPLPADDFEDLNPPKK
NCU09435.1	EQQQPQLPMSQRDTPAAGFSPR-PQGLKSPSAGPGAPFAPYAAKRPLDDRDYDDH--PRK
FG00349.1 	QHQHQQS-------ISLRNSPMPVRQEQNPRPQ--YVRATASPKRPLAVDD-EELNPPKR
AN4892.1  	QVP------------SRHGTPS----SRFPEAS-----VTNSPKRPLEDFD-DDMSRPRK
          	:              :   :*      . *        .  :.****   * :: . *::

MG03265.1 	IQRNDQREFQRAESPLKGAAGRR-LENQRRIHGQGPASYNTAP------APAIPREINFL
NCU09435.1	IARSEHDPFVRGASPLKGAAGRR-LDQQRRMGGAAGAYSGSGAG---SQVAPIARDITFL
FG00349.1 	LAR--------GASPLKGAAGRR-LDQQRRNQ-----------------ASALHRDITFL
AN4892.1  	FVR--------GESPLRTTTQRRQLDQQKRTQTALQVPSSGSQYRSQGSPAPLPRDIVYL
          	: *        . ***: :: **.*::*:*         . .   :... ..: *:* :*

MG03265.1 	LSQLPGAEVYNSTRLSASRVVDTLRDTHVPDYQSWKSRQDKGLRQSGAQMPSDFSNPGYG
NCU09435.1	LSQIPRVEFYDSHRLNPSRMVSLLQNVKVPEYLDWKRERER--MQQMQQMQGDGYG-GYG
FG00349.1 	LNILPSSQSYDAQRFNSAALVSILRDTEIPDFATLK---------------------AAG
AN4892.1  	LSVIPPASTYTAGRFSAEKMIDLVRRLDLPASISQIPLPQS--------------ARGLG
          	*. :*  . * : *:..  ::. ::  .:*         .                 . *

MG03265.1 	RDSPGLRTGSPFGG--ERRIAS~ASSTYQRPASRDSYEPPPPSG---YQPPSATLQYPPA
NCU09435.1	GGGGGGGGGGYQGGGHARNISQ~DYAYRESPGPLGGRPLSPFTGGPGSRLASATAAYRQA
FG00349.1 	GGQP--------------RFGN~PTHTRQPSGEFVNRPLSPYGR-----MSAAAGGYRNS
AN4892.1  	TGQTTIGG---------QQFSD1DGGYYYKGGVVDMCALHFMDDGVFRLYVAGLAGNILA
          	 .                .:..         .            .      :.      :

MG03265.1 	TPSYDNQPTHGAWPPPPHQLPYGAPPPAQNYAQAPPQLYQTPTGQQQAQP--PYG--TYR
NCU09435.1	P--VGRPESSGSYEPPP-AAQYGVPPPAQYDGGWAQQQQQQQYGQPPAPQGYRYGNPPPP
FG00349.1 	P---LRPETGNAYQSNP------YPPPEASGQQPTWPQAPGGYGAPAPG---QFG--GYR
AN4892.1  	R-----------------------ILFFGSGGLLAYLLRTVQIWDPEIPRR---------
          	                                  .                         

MG03265.1 	Y
NCU09435.1	Y
FG00349.1 	Y
AN4892.1  	-
          	
```
